# Melittin-Related Peptides Interfere with Sandfly Fever Naples Virus Infection by Interacting with Heparan Sulphate

**DOI:** 10.3390/microorganisms11102446

**Published:** 2023-09-29

**Authors:** Annalisa Chianese, Carla Zannella, Francesca Palma, Laura Di Clemente, Alessandra Monti, Nunzianna Doti, Anna De Filippis, Massimiliano Galdiero

**Affiliations:** 1 Department of Experimental Medicine, University of Campania “Luigi Vanvitelli”, 80138 Naples, Italy; annalisa.chianese@unicampania.it (A.C.); carla.zannella@unicampania.it (C.Z.); francesca.palma@unicampania.it (F.P.); diclementelaura.ldc@gmail.com (L.D.C.); anna.defilippis@unicampania.it (A.D.F.); 2 Institute of Biostructures and Bioimaging (IBB), National Research Council (CNR), 80131 Naples, Italy; alessandra.monti@ibb.cnr.it (A.M.); nunzianna.doti@cnr.it (N.D.); 3 Section of Virology and Microbiology, University Hospital of Campania “Luigi Vanvitelli”, 80138 Naples, Italy

**Keywords:** phlebovirus, arbovirus, sandfly-borne infections, heparan sulfate, glycosaminoglycans, sandfly fever Naples virus, Toscana virus, Crimea–Congo hemorrhagic fever, Rift Valley fever virus

## Abstract

Emerging viruses pose an important global public health challenge, and early action is needed to control their spread. The *Bunyaviridae* family contains a great number of arboviruses which are potentially pathogenic for humans. For example, phleboviruses affect a large range of hosts, including humans and animals. Some infections usually have an asymptomatic course, but others lead to severe complications, such as Toscana virus, which is able to cause meningitis and encephalitis. Unfortunately, to date, no vaccines or antiviral treatments have been found. In the present study, we evaluated the effect of melittin-related peptides, namely the frog-derived RV-23 and AR-23, on sandfly fever Naples virus infection in vitro. Both peptides exhibited a strong antiviral activity by targeting the viral particles and blocking the virus–cell interaction. Their action was directed to an early phase of SFNV infection, in particular at viral adsorption on host cells, by interfering with the binding of common glycosaminoglycan receptors. Given the better antimicrobial behavior of AR-23 and RV-23 compared to melittin in terms of selectivity, our studies expand our understanding of the potential of these peptides as antimicrobials and stimulate further investigations in the direction of novel antiviral strategies against phlebovirus infection.

## 1. Introduction

An increase in the spread of viral infections causing diseases and epidemics has been observed in recent years. New or re-emerging viruses have often caught the scientific community unexpectedly, without adequate preparation to counter them, as demonstrated by the coronavirus disease 2019 (COVID-19) pandemic. Arthropod-borne viruses play an important role among emerging and re-emerging infectious diseases and, although some infections can lead to mild flu-like symptoms, most develop into severe forms of meningitis and meningoencephalitis [1]. Humans become infected through arthropod vectors, contaminated body fluids, or via rodent feces, like in the case of hantavirus. Among arboviruses belonging to the *Phlebovirus* genus, sandfly-borne phleboviruses may cause a transient febrile illness (sandfly fever) or more severe neuroinvasive disease [2,3,4,5]. In many European countries, phleboviruses have caused epidemic human outbreaks, and new species, previously confined to tropical and sub-tropical regions, have become endemic [6,7,8,9]. Toscana virus infection has spread in the Mediterranean basin, including Italy, producing asymptomatic or pauci-symptomatic infections but, due to its neuroinvasive tropism, rarely is the infection characterized by severe neurological complications. For these reasons, since 2018 the Italian National Health Service has included Toscana virus infections in the surveillance for human arboviral infections. Also, other phleboviruses can cause severe diseases in humans, such as Rift Valley fever virus (RVFV), severe fever with thrombocytopenia syndrome virus (SFTSV) and Crimean–Congo hemorrhagic fever virus (CCHFV), which are able to lead to hemorrhagic fever [10,11,12,13]. Despite the increasing impact of sandfly fever diseases on human and animal health, there are no available approved therapies or prophylactic treatments to date.

Antimicrobial peptides (AMPs) are emerging as a valid alternative for the cure of infectious diseases, gaining notable attention in research as well as in the pharmaceutical area. The peptides are evolutionarily conserved among various organisms as a part of the innate immune system. They can derive from a plethora of sources and are short peptides consisting of 10–50 amino acids. Their popularity lies in their small size and ease of synthesis, and they are associated with low host toxicity, high selectivity, potency, and efficacy. They are different from small molecule drugs in that AMPs can act on a wide range of targets and, therefore, drug resistance phenomena are less common. In detail, AMPs with antiviral activity, alternatively regarded as antiviral peptides (AVPs), mainly show a virucidal effect by directly inhibiting the viral particles [14,15,16,17], as we recently demonstrated with temporin L and its analogues, which are able to block the viral infection of a large group of enveloped viruses by totally destroying the viral surfaces [14]. Other mechanisms by which AVPs exert their inhibitory activity involve the interference with cell receptors deputed to the fusion/entry of the virus in the host [18] or internal target [19] or through the blocking of viral gene expression [20].

Melittin (primary sequence: GIGAVLKVLTTGLPALISWIKRKRQQ) is a natural peptide derived from the venom of the European honeybee *Apis mellifera* with well-known antimicrobial properties [21,22,23,24,25] due to its ability to form pores in membranes and lyse the cell. Regarding the antiviral activity, melittin is able to act on a wide variety of viruses, including arenaviruses [26], herpesviruses [26,27], flaviviruses [28], picornaviruses [29], influenza viruses, and paramyxoviruses [30], as well as against the human immunodeficiency virus type 1 (HIV-1) [31,32]. Its main action is directed on viral envelopes or capsid proteins, interfering with the viral entry process. Furthermore, other potential targets may be the reduction of viral mRNA inside the infected cell [30], deactivation of viral packaging [33], and inhibition of viral-cell membranes fusion [34]. However, the main drawback of this peptide is its low selectivity, as evidenced by its ability to permeabilize different types of membranes resulting in toxicity to human cells [35]. Two melittin-related peptides were later found in frogs, namely AR-23, derived from *Rana tagoi* [36], and RV-23, from *Rana draytonii* [37]. The first peptide (AR-23: AIGSILGALAKGLPTLISWIKNR-NH_2_) is very similar to melittin (78% of identities in the primary sequence), while the second (RV-23: RIGVLLARLPKLFSLFKLMGKKV-NH_2_) has the same content of leucine and isoleucine residues, and a net charge (+7) similar to melittin (+5). AR-23 and RV-23 showed similar antibacterial activity and significantly reduced cytotoxicity effects compared to melittin, thus resulting in better antimicrobial compounds for therapeutic purposes [38]. In detail, both peptides exhibited lytic activity against *Staphylococcus aureus* and *Escherichia coli*, with RV-23 showing the highest effect [38]. On the contrary, less is known about AR-23 and RV-23 antiviral activities. Our previous data indicated that AR-23 had a broad-range antiviral effect against enveloped viruses [15]. The aim of the present study was to explore the antiviral potential of AR-23 and RV-23, especially the effect of these peptides against the sandfly fever Naples virus (SFNV), used as surrogate virus for most pathogenic viruses of the same family. Our results indicate that both melittin-related peptides specifically inhibit viral cytopathic effect and that their action is directed in an early phase of infection. In detail, AR-23 and RV-23 are able to interfere with the viral infection by binding to glycosaminoglycans (GAGs), the cell receptors used by the virus for the entry into the host cells.

## 2. Materials and Methods

### 2.1. Peptide Synthesis and Characterization

C-terminal amidated RV-23 and AR-23 were synthesized by the optimized Fmoc solid-phase peptide synthesis protocol [15]. In addition, two scramble peptides, namely FGILAKSVVRPKGRKFLKLMLLL-NH_2_ and IKWLNISAATGILGRIALKSGPL-NH_2_, were synthesized as negative controls of RV-23 and AR-23, respectively. Their sequences were obtained using the peptidenexus tool (https://peptidenexus.com/article/sequence-scrambler, accessed on 14 September 2023). Protected amino acids, coupling agents (HATU, Oxyma) and Fmoc-Rink Amide AM resin, solvents, i.e., acetonitrile (CH_3_CN), dimethylformamide (DMF), trifluoroacetic acid (TFA), symcollidine, diisopropylethylamine (DIPEA), and piperidine were acquired from Merck (Milan, Italy). High Performance Liquid Chromatography (HPLC) was executed by a WATERS 2545 preparative system (Waters, Milan, Italy) fitted with a WATERS 2489 UV/Visible detector, applying a linear gradient of CH_3_CN/0.05%TFA in water 0.05% TFA from 5 to 70% in 20 min at a flow rate of 12 mL/min. Peptides Mass spectrometry (MS) characterization was carried out by using an ESI-TOF-MS Agilent 1290 Infinity LC System coupled to an Agilent 6230 time-of-flight (TOF) LC/MS System (all equipment was supplied by Agilent Technologies, Cernusco sul Naviglio, Italy). The LC Agilent 1290 LC module was coupled with a photodiode array (PDA) detector and a 6230 time-of-flight MS detector, along with a binary solvent pump degasser, a column heater, and an autosampler. Liquid chromatography (LC)-MS characterization was performed using a C18 Waters xBridge column (3 µm, 4.6 × 5.0 mm), applying a linear gradient of CH_3_CN/0.05% TFA in water 0.05% TFA from 5 to 70% in 20 min at a flow rate of 0.2 mL/min. The yields of target peptides, calculated as ((experimental weight of pure peptide)/(theoretical weight) × 100), where the theoretical weight was calculated based on the synthesis scale used, were estimated to be about 70%. The relative purity of peptides was calculated as the ratio of peak area of the target peptide and the sum of areas of all detected peaks from the UV chromatograms at 210.4 nm. The purity was >98%. Peptides stability was performed in the cell culture medium by following this protocol: peptides were diluted in medium to a final concentration of 1.0 mg/mL and incubated at 37 °C. Aliquots of 50 µL of each peptide after 0, 30, 60, and 120 min were collected and analyzed by RP-HPLC on a C18 Onyx column (4.6 × 50 mm, 5 µm), applying a linear gradient from 5 to 70% of solvent B (0.1% TFA in CH_3_CN) over solvent A (0.1% TFA in H_2_O) in 10 min at a flow rate of 0.6mL/min. All stability tests were performed at least in triplicate. Peptide concentrations in solution were determined from RP-HPLC peak areas compared to peak areas obtained at t0 (0 min control set to 100% for each peak). The results obtained showed that the peptides are stable in our experimental conditions (see Supplementary Figure S1).

### 2.2. Cells and Viruses

Vero cells (ATCC CRL-1587) were grown at 37 °C and 5% CO_2_ in Dulbecco’s modified Eagle’s medium (DMEM; Microtech, Naples, Italy) with the addition of 10% inactivated fetal bovine serum (FBS, Microgem, Naples, Italy), 2 mM glutamine, and 1X antibiotic solution (penicillin/streptomycin solution). SFNV strain UVE/SFNV/UNK/IT/30451 (acquired by the European virus archive global, EVAg) was propagated in Vero cells and titrated by median tissue culture infectious dose (TCID50). In brief, the virus was serially diluted and incubated in 96-well plates containing 2 × 10^5^/mL adherent Vero cells seeded the previous day. Plates were incubated at 37 °C for 5 days prior evaluation of CPE via microscope and were then fixed and stained with Gram’s crystal violet solution.

### 2.3. Cytotoxicity Assay

Confluent Vero cells were treated with twofold serial dilutions of each peptide (50, 25, 12.5, 6.25, 3.125 μM). The 96-well plates were incubated at 37 °C/5% CO_2_ for 24 h; then, cell viability was analyzed by the 3-(4,5-Dimethylthiazol-2-yl)-2,5-Diphenyltetrazolium Bromide (MTT, Sigma-Aldrich, St. Louis, MO, USA) assay based on the reduction of the yellowish MTT to dark blue formazan salts. In brief, at the end of incubation, 100 μL of MTT solution (5 mg/mL) was incubated in each well for 3 h at 37 °C. Supernatant was removed and 100 μL of DMSO 100% (Sigma Aldrich) was added to dissolve formazan. Cytotoxicity was evaluated by spectrophotometric reading at 540 nm. Cell viability was calculated as a percentage of control cells. All experiments were performed in triplicate and means and standard deviations were reported. Nonlinear regression analysis was performed using GraphPad Prism software (version 8.0.1) to extract the CC_50_.

### 2.4. Antiviral Assays

To analyze whether peptides were endowed with antiviral activity against SFNV, and, specifically, what stage of the infection was affected, different experimental procedures were carried out: (i) co-treatment assay: the cells (5 × 10^5^/mL) were incubated with each peptide (at non-cytotoxic concentrations) and infected with the virus (200 TCID50/mL) at the same time (1 h at 37 °C); (ii) virus pre-treatment assay: peptide was added together with the virus inoculum during the adsorption step (1 h at 37 °C) and the mixture was subsequently titrated on cells; (iii) cell pre-treatment assay: cells were previously incubated with the peptide and then infected (1 h at 37 °C); (iv) post-treatment assay: peptide was incubated with the cells after the viral adsorption step (1 h at 37 °C). Following the incubation, the cell monolayer was rinsed three times with medium and than supplemented with DMEM 5% FBS. After 5 days post-infection, cythopathic effect (CPE) was observed and cells were stained with 0.5% crystal violet. All experiments were performed in triplicate. The inhibition rate of the infectivity was evaluated by CPE observed in the wells treated with the peptides to CPE in positive control (cells infected with virus, without peptide).

### 2.5. Heparin Assay

To analyze if heparin could neutralize the virus binding to cells, Vero were infected with SFNV (200 TCID50/mL) and simultaneously treated with serial heparin dilutions (1.56, 6, 25, 100, 400, and 1600 μg/mL) for 1 h at 37 °C. Then, the cell monolayer was washed with medium and incubated in the presence of DMEM 5% FBS for 5 days until the CPE appeared. To analyze the simultaneous effect of heparin and each peptide against SFNV infection, a standard concentration of RV-23 or AR-23 (10 μM) was incubated together with the same above-mentioned heparin concentrations. At the same time, the virus (200 TCID50/mL) was added to cell monolayer and subsequently incubated for 1 h at 37 °C. The infection was observed after 5 days infection via CPE.

### 2.6. Circular Dichroism (CD) Measurements

The CD spectra of the peptide–heparin complexes were recorded from 260 to 190 nm, using the JASCO-705 CD spectrophotometer (Jasco International Co. Ltd., Tokyo, 130 Japan) at room temperature. All spectra were recorded using a 0.1 cm path length cuvette with a scan speed of 50 nm/min, response time of 1 s, and bandwidth of 1 nm. Each spectrum was collected by averaging 3 spectra. Sample solutions were prepared using 5.0 mM sodium phosphate buffer at pH 7.4. The peptides at a fixed concentration of 50 μM were titrated with increasing concentrations of heparin (1, 5, 10, and 25 μM). Each spectrum was corrected by subtracting the heparin spectrum without the peptides at the appropriate concentration. Graphs were prepared using GraphPad Prism 5.1 software (GraphPad Software, 149 San Diego, CA, USA).

### 2.7. Statistical Analysis

Statistical analysis was performed by one-way ANOVA followed by Dunnett’s multiple comparisons test calculated using GraphPad Prism, version 8.0.1. Data were expressed as the mean and SD, and *p* values of < 0.05 were considered significant.

## 3. Results

### 3.1. Cytotoxicity of RV-23 and AR-23 on Vero Cells

**Figure 1:**
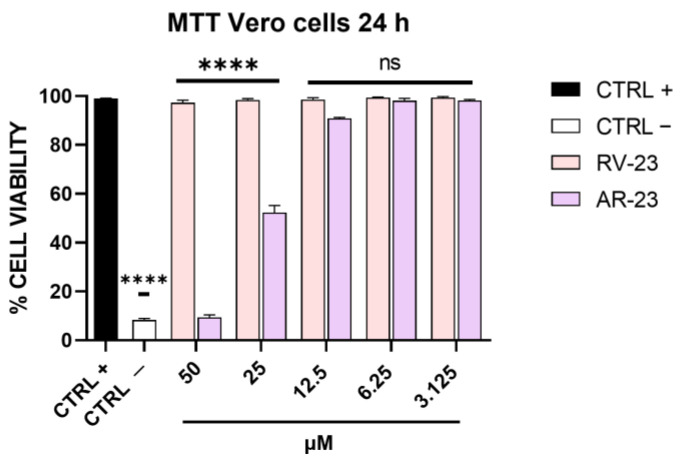
Cytotoxicity analysis after RV-23 and AR-23 treatment. Vero cells were treated with each peptide in the range of concentrations from 50 to 3.125 μM. After 24 h, MTT assay was carried out. Cell viability was calculated as a percentage of control cells. Positive control (CTRL +) indicates non-treated cells, i.e., cells with culture medium; negative control (CTRL −) refers to cells treated with dimethyl sulfoxide (DMSO) able to kill cell monolayer. **** *p* < 0.0001; ns: non-significant.

A preliminary experiment was carried out to evaluate the maximal non-cytotoxic concentration of RV-23 and AR-23. Twofold serial dilutions of each peptide were incubated with Vero cells for 24 h at 37 °C. Then, cell viability was determined via MTT assay, and as indicated in Figure 1, RV-23 was not cytotoxic up to the highest dose. On the other hand, AR-23 consistently affected cell viability at 50 μM, and the 50% cytotoxic concentration was at 25 μM [15]. These results set the peptide concentration range to be used in the subsequent assays: from 50 μM to 3.125 μM for RV-23 and from 25 μM to 3.125 μM for AR-23.

### 3.2. Effect of RV-23 and AR-23 on Different Steps of Viral Infection

**Figure 2:**
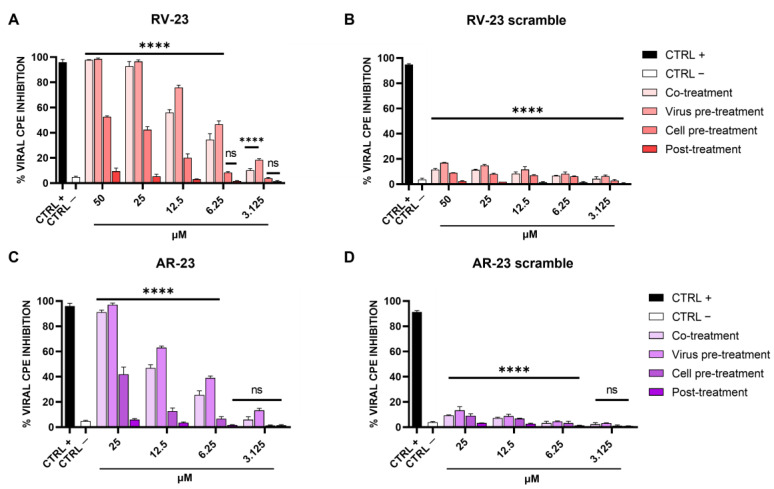
Effect of RV-23, AR-23, and their scramble peptides on different steps of SFNV infection. Different schemes were performed by treating Vero cells with (**A**) RV-23, (**B**) RV-23 scramble, (**C**) AR-23, or (**D**) AR-23 scramble. In co-treatment, wells received peptide and virus inoculum simultaneously, incubated for 1 h, then media was replaced, and cells were incubated. For virus pre-treatment, the virus was incubated with peptide for 1 h, and then diluted to obtain ineffective peptide concentrations and added to cells. For cell pre-treatment, each peptide was incubated with cells for 1 h, media was then removed, and virus inoculum added and incubated for 60 min; then inoculum was discarded and replaced with fresh medium and cells were incubated. In post-treatment, wells were infected with virus for 1 h followed by inoculum removal and replacement with peptide in the media. CPE was observed after 5 days of infection by crystal violet staining. CTRL + refers to ribavirin-treated cells (100 μM) [39]; CTRL—are infected cells with no treatment. **** *p* < 0.0001; ns: non-significant.

To investigate if melittin-related peptides had an antiviral effect against SFNV and whether the effect affected viral adsorption or other steps of viral replication, the inhibitory activities of RV-23 and AR-23 were analyzed using different experimental approaches: (i) co-treatment: simultaneous addition of peptide and virus on cell monolayer; (ii) virus pre-treatment: pre-incubation peptide–virus and mixture dilutions on cells to reach a non-cytotoxic peptide concentration; (iii) cell pre-treatment: infection on peptide pre-treated cells; and (iv) post-treatment assays: peptide treatment after viral infection. The difference between the assays was the timing of incubation of peptides on the cell monolayer [14]. The respective scramble peptides were used as negative controls. The results are shown in Figure 2, and they reveal a putative action of RV-23 and AR-23 in a very early stage of SFNV infection.

In detail, both peptides exhibited the best antiviral performance when they were added together with the virus on Vero cells (co-treatment) and, above all, when they were pre-incubated with the virus and then titrated on cells (virus pre-treatment). These results highlighted the peptides’ inhibitory role during the adsorption phase of the SFNV infectious cycle. RV-23 was found to be more active than AR-23, with an inhibitory concentration of 50% (IC_50_) at 10 μM and 12.5 μM in co-treatment and 6.25 μM and 10 μM in virus pre-treatment assays. In addition, a moderate activity was detected in cell pre-treatment, when peptides were incubated on cells prior the infection, suggesting a putative action of RV-23 and AR-23 on the cell surface that interferes with the virus–cell binding. On the contrary, no activity was observed after the viral adsorption step (post-treatment), indicating that the peptides did not interfere later in the infection. Using the data obtained from RV-23 and AR-23 scramble peptides (Figure 2B,D), we can hypothesize that the antiviral activity of the two melittin-related peptides was strongly associated with the peptide primary sequence and with the interaction between peptide positive residues and the negatively charged cell membranes.

### 3.3. Neutralization of SFNV Infection by Heparin and Peptides

**Figure 3:**
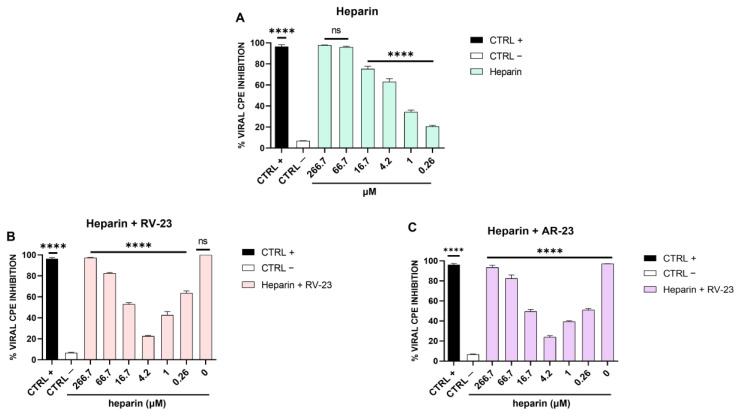
Heparin assay. (**A**) SFNV infection was neutralized in the presence of heparin (in the range of concentration from 266.7 to 0.26 μM). (**B**) Effect of heparin and RV-23 or (**C**) AR-23 in combination against SFNV infection. Serial heparin concentrations were incubated with the virus and each peptide (25 μM) on Vero cells, and after 5 days of infection, CPE was analyzed by crystal violet staining. CTRL + refers to ribavirin-treated cells (100 μM) [39]; CTRL—are infected cells with no peptide. **** *p* < 0.0001; ns: non-significant.

Our findings demonstrated that the antiviral activity is principally addressed to viral attachment on the host cells. Phleboviruses use GAGs for the viral entry [40], including heparin and heparan sulphate (HS). Therefore, we assumed that if SFNV also engages GAGs for the attachment and entry into the target cells, the antiviral effect of RV-23 and AR-23 peptides could be due to a competition with the virus for the same cellular receptors. In this context, we first explored whether heparin could interfere with SFNV infection. Different concentrations of heparin were incubated with the virus during the adsorption phase, and after 5 days of infection, the CPE was analyzed (Figure 3A).

We observed a reduction in the viral infection in a dose-dependent manner, indicating that heparin expressed on the cell surface is involved in the SFNV adsorption phase. Then, we investigated whether RV-23 and AR-23 could compete with heparin for the binding to the host cells. Heparin at different concentrations and each peptide (25 μM) were added on Vero cells simultaneously to viral infection. At 5 days from the infection, the % of CPE inhibition was measured by crystal violet staining (Figure 3B,C). At the highest doses of heparin (266.7 and 66.7 μM), the antiviral activity of both peptides was due mainly to the heparin since the peptides did not consistently influence the % viral CPE inhibition. Once the heparin concentration was reduced, as particularly evidenced at 4.2 μM, the inhibitory activity of peptides decreased. Finally, at low doses of heparin (1 and 0.26 μM), the inhibitory effect of the peptides was increased.

**Figure 4:**
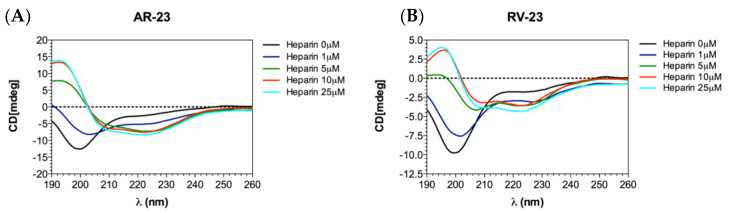
CD analysis. CD measurements of AR-23 (**A**) and RV-23 (**B**) titrated with heparin. Each spectrum was corrected by subtracting the heparin spectrum without the peptides at the appropriate concentration.

We also evaluated the conformation of the peptides in the absence and presence of heparin via CD measurements. The data obtained demonstrated that both peptides interacted with heparin. As observed in Figure 4, both peptides alone in solution showed a strong negative peak at approximately 198 nm, corresponding to a highly flexible conformation. However, adding increasing amounts of heparin to the peptides resulted in a dose-dependent conformational change. In fact, the appearance of two negative peaks at 208 and 222 nm and a positive band at 190 nm was observed, characteristic of an alpha-helical conformation.

## 4. Discussion

Vector-borne diseases account for 700,000 deaths every year [41], of which most are transmitted by arthropods, including mosquitos, sand flies, and ticks, living in different habitats and affecting both urban and rural populations, and humans and animals. The last deadly viral outbreaks originated from these vectors, such as California encephalitis, chikungunya, dengue, eastern equine encephalitis, Powassan, St. Louis encephalitis, West Nile, Yellow Fever, and Zika. In Italy, after the eradication of malaria in the 1950s, vector-borne diseases have been transmitted by ticks and sand flies. The current issue is that some of these diseases, often restricted to a few foci, are spreading in totally new areas.

The most important species of sandfly-borne phleboviruses is the Toscana virus, able to infect central and peripheral nervous systems causing meningitis and encephalitis [9,42]. Even if other infections are asymptomatic, or generally associated with flu-like symptoms, new pheleboviruses are continuously found, evidencing a possible eruption of diseases not yet reported in humans. This is due mainly to the segmented genome of phleboviruses, which favors the recombination phenomenon, increasing the expansion of viral host ranges and tissue tropism and exacerbating virulence and pathogenesis [43,44,45]. Therefore, the control and containment of arthropod-borne infections are mandatory for public health. However, to date, there are no available vaccines or therapies to fight these diseases.

In the present study, we evaluated the activity of two melittin-derived peptides, namely AR-23 and RV-23, against SFNV infection. Our findings demonstrated that both of the peptides were able to interfere with the viral infection in a dose-dependent manner (Figure 2). Regarding the specific stage of the SFNV lifecycle, the peptides consistently inhibited the viral attachment and, in part, the virus–cell interaction (Figure 2). RV-23 exhibited the best performance, with an IC_50_ at 6.25 μM when incubated directly on viral particles; on the other hand, AR-23 was characterized by an IC_50_ at 10 μM in the same treatment. These data agree with our previous study [15], in which we analyzed the AR-23 antiviral effect against a large panel of enveloped viruses, such as herpes simplex virus type 1 (HSV-1), several paramyxoviruses, and coronaviruses, including the severe acute respiratory syndrome coronavirus type 2 (SARS-CoV-2). We observed that AR-23 was able to target the viral envelope in an early phase of infection, destroying it with a mechanism shared among all the viruses tested. The present study improved our knowledge on this peptide. By adding a similar peptide to the study, i.e., RV-23, which has greater antiviral potential and a lower cytotoxic profile, we deepened our understanding of the peptide mechanism of action.

We observed that the peptides had two targets which merged into a single antiviral effect, namely the viral entry inhibition. As already demonstrated [14,15], AMPs have a virucidal action, but we found for the first time that they are able to intercept the cell surface by binding to host cell receptors. Bunyaviruses have two glycoproteins on their surface, namely Gc and Gn, mediating the entry into the target cell. Cell receptors allowing bunyavirus entry are not fully elucidated. La Crosse virus uses DC-SIGN as a cellular receptor [46], while CCHFV binds to integrins and nucleolin [47] as do some hantaviruses [48]. On the contrary, it has been widely reported that GAGs are common receptors that viruses use for their productive entry, including herpes simplex virus [49,50], adenovirus [51,52], respiratory syncytial virus [53,54], human papillomavirus [55], foot-and-mouth disease virus [56], hepatitis B virus [57,58,59], hepatitis C virus [60,61], Ebola virus [62], dengue virus [63], HIV [64,65], and others [66,67,68,69,70]. Among phleboviruses, Rift Valley fever virus (RVFV) requires heparan sulfate for an efficient infection [71,72,73]. It was recently demonstrated that RVFV entry was compromised by pre-incubating the virus with heparin, or by enzymatic removal of heparan sulfate from cells and in cells genetically deficient in heparan sulfate synthesis [73]. In this scenario, we investigated first whether heparan sulfate is involved in SFNV infection by infecting Vero cells in the presence of different concentrations of heparin, used as an analog of heparan sulphate (Figure 3A). Heparin strongly blocked the viral infection up to 4.2 μM, indicating that SFNV entry into the host cell was GAG-dependent. We further investigated if RV-23 and AR-23 could compete with heparin for heparan sulfate binding. Then, different concentrations of heparin were incubated on Vero cells in the presence of 25 μM of the peptides, and cells were infected with SFNV during the viral adsorption step (Figure 3B,C). We observed a strong reduction of the antiviral activity of both peptides when mixed together with heparin. In detail, the antiviral effect was partially abolished when RV-23 (Figure 3B) and AR-23 (Figure 3C) were added with 4.2 μM of heparin, indicating that the compounds were able to interact with the same target. Our results showed that peptides could bind to heparan sulfate, inhibiting the initial step in the interaction between the virus and the cell surface. We hypothesized that the cationic nature of the peptides could influence the interaction with negatively charged sulfated and carboxylated groups of heparan sulfate. This hypothesis could also justify the higher activity of RV-23 with respect to AR-23, since the former is rich in lysine and arginine residues (net charge: +6) which promote a stronger interaction with GAGs on the cell surface. In addition, the highly selective antiviral activity and reduced cytotoxicity of RV-23 may be partly due to its conformation. Indeed, it has already been demonstrated that melittin, RV-23, and AR-23 were structured in α-helix, and that RV-23 was characterized by the lowest α-helix content and hydrophobicity, as well as the highest hydrophobic moment [38]. Since hydrophobicity and α-helix content are strongly associated with peptide toxic profile, our results could explain the better performance of RV-23 with respect to AR-23 and the related-melittin peptide.

## 5. Conclusions

In summary, our findings deepen the knowledge about the mechanism of action of melittin-related peptides against viral infection. Both of the peptides (in particular RV-23, due to its strong cationic nature, lower α-helix content, and hydrophobicity) are able to block SFNV entry in the target cells through the binding and blocking of GAG receptors. In detail, heparan sulfate can bind to different ligands, including growth factors, cytokines, chemokines, enzymes, and bacterial and viral pathogens. Heparan sulfate is expressed on a variety of cells and tissues, highlighting its valuable role as a target for the therapy of many human diseases. Our results are promising for the development of a novel antiviral approach against not only SFNV infection but, in general, against infections caused by other phleboviruses which are potentially pathogenic for humans.

## Data Availability

The data presented in this study are available on request from the corresponding author. The authors can confirm that all relevant data are included in the article.

## Supplementary Materials

The following supporting information can be downloaded at: https://www.mdpi.com/article/10.3390/microorganisms11102446/s1, Figure S1: Peptide stability profiles in DMEM. Peptide concentrations in solution were determined from RP-HPLC peak areas compared to peak areas obtained at t0 (0 min control set to 100% for each peak).
