# Supplementary material for: Melittin-Related Peptides Interfere with Sandfly Fever Naples Virus Infection by Interacting with Heparan Sulphate
Source: Microorganisms. 2023 Sep 29;11(10):2446. doi: 10.3390/microorganisms11102446 (PMC10609114; doi:10.3390/microorganisms11102446)
Supplement: Supplementary file 1 [file microorganisms-11-02446-s001.zip › microorganisms-2560592-supplementary.pdf]

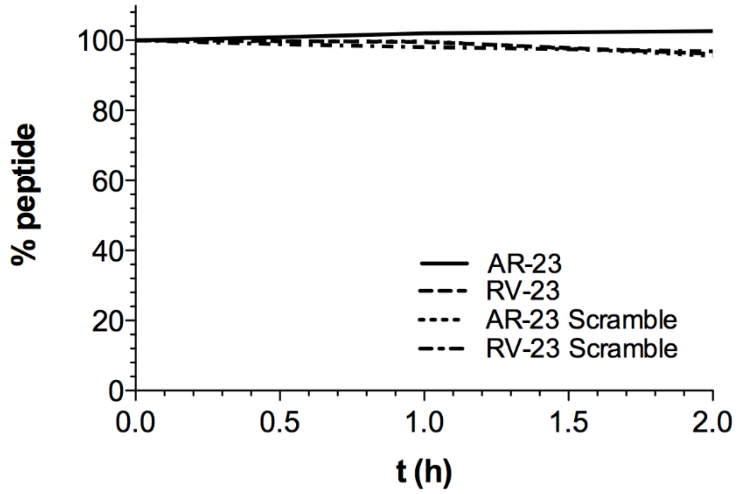

Figure S1. Peptide stability profiles in DMEM. Peptide concentrations in solution were determined from RP-HPLC peak areas compared to peak areas obtained at t0 (0 min control set to 100% for each peak).
